# A SAW‐Based Programmable Controlled RNA Detecting Device: Rapid In Situ Cytolysis‐RNA Capture‐RNA Release‐PCR in One Mini Chamber

**DOI:** 10.1002/advs.202309744

**Published:** 2024-05-21

**Authors:** Yupeng Yang, Zenan Wang, Hetao Xie, Ying Hu, Hong Liu

**Affiliations:** ^1^ Shenzhen Institute of Advanced Technology Chinese Academy of Sciences Shenzhen 518000 P. R. China; ^2^ University of Jinan Jinan 250022 P. R. China

**Keywords:** microfluidic, RNA detection, surface acoustic wave

## Abstract

Viral RNA detection is crucial in preventing and treating early infectious diseases. Traditional methods of RNA detection require a large amount of equipment and technical personnel. In this study, proposed a programmable controlled surface acoustic wave (SAW)‐based RNA detecting device has been proposed. The proposed device can perform the entire viral RNA detection process, including cell lysis by cell‐microparticle collision through SAW‐induced liquid whirling, RNA capture by SAW‐suspended magnetic beads, RNA elution through SAW‐induced high streaming force, and PCR thermal cycling through SAW‐generated heat. The device has completed all RNA detection steps in one mini chamber, requiring only 489 µl reagents for RNA extraction, much smaller than the amount used in manual RNA extraction (2065 µl). The experimental results have shown that PCR results from the device are comparable to those achieved via commercial qPCR instrumental detection. This work has demonstrated the potential of SAW‐based lab‐on‐a‐chip devices for point‐of‐care testing and provided a novel approach for rapidly detecting infectious diseases.

## Graphical abstract

This study proposes a new approach for viral RNA detection using a programmable controlled surface acoustic wave‐based device. It can perform all steps for RNA detection in a single mini chamber, requiring only 489 µl of reagents. This novel approach demonstrates the potential of SAW‐based lab‐on‐a‐chip devices for point‐of‐care testing for infectious diseases.

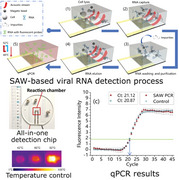

## 1 Introduction

Rapid detection of infectious diseases can play a decisive role in the early prevention like COVID‐19 and treatment of the disease.^[^ ^1^ ^]^ Traditional viral RNA detection requires expensive and complicated equipment, which will cause a tremendous medical burden in large‐scale infectious diseases.^[^ ^2^, ^3^ ^]^ Highly miniaturized microfluidic chips, which integrate complex fluid manipulation systems and functional unit modules for sample preparation, reaction, sorting, detection, and other basic operations on a chip of a few square centimeters, provide an potential tool for the integration of complex viral RNA detection processes.^[^ ^4^ ^]^ It has the characteristics of small volume, less composition, high efficiency, automation, and integration.^[^ ^5^, ^6^ ^]^

Viral RNA detection includes RNA extraction and polymerase chain reaction (PCR), the basic RNA extraction process includes cell lysis, RNA capture, purification, and release.^[^ ^7^ ^]^ At present, RNA extraction based on a microfluidic chip has been developed.^[^ ^7^, ^8^, ^9^ ^]^ However, most of the current microfluidic viral RNA extraction based on chemical cell lysis^[^ ^10^, ^11^ ^]^ or biological cell lysis.^[^ ^12^, ^13^ ^]^ These methods may cause many reagent residues, which will have great difficulties in subsequent processing on the microscale. In terms of viral RNA detection, the combination of microfluidic chips and PCR has become a research hotspot, and different types of microfluidic PCR chips have been applied in various fields.^[^ ^14^, ^15^, ^16^, ^17^ ^]^ Efficient reaction heaters that perform heating cycles are essential for microfluidic PCR chips. Reaction heaters are generally categorized into contact and non‐contact types.^[^ ^18^ ^]^ Typical contact heaters include thin film, heated metal blocks, Peltier^[^ ^19^ ^]^, and so on. These heaters are low‐cost and have high heating rates, but their cycle efficiency is affected by high heat loss. Non‐contact heaters, such as infrared heating^[^ ^20^ ^]^ and microwave heating,^[^ ^21^ ^]^ are more versatile in integrating and facilitating PCR microfluidic chip construction.

Acoustofluidics based on surface acoustic waves (SAW) has recently gained much attention in the research field of lab on a chip.^[^ ^22^ ^]^ Since the energy and momentum carried by the SAW during the formation and propagation process can be precisely controlled through various designs, such as different interdigital transducer (IDT) designs and material selections,^[^ ^23^, ^24^ ^]^ SAW has been widely used in biological sample manipulation and analysis.^[^ ^25^, ^26^ ^]^ Wang et al. developed a SAW‐based lysis chip with a 3‐inch piezoelectric material to achieve efficient different cell lysis.^[^ ^27^ ^]^ However, they did not accomplish the extraction and evaluation of RNA. Additionally, the lysis operation was carried out in an exposed environment. Considering that RNA is highly susceptible to degradation in air, this method carries an extremely high risk. Reboud et al. successfully employed SAW to achieve reagent‐free cell lysis, thermal cycling for PCR, and detection of parasite DNA.^[^ ^28^ ^]^ However, the critical steps of DNA detection, including DNA extraction, DNA purification and PCR sample pretreatment, were all performed manually. They did not achieve a “sample‐to‐answer” process for virus detection. In addition, further research is needed to evaluate the RNA viruses that are susceptible to degradation, such as COVID‐19, since the PCR achieved by this method was based on relatively stable DNA. Zhao et al. developed a SAW‐based centrifugal microfluidic chip with full integration of RNA extraction and detection;^[^ ^8^ ^]^ Salman et al. developed a shunting microfluidic PCR device for rapid bacterial detection.^[^ ^29^ ^]^ Microfluidic chips for nucleic acid detection usually use traditional biological or chemical lysis methods. However, traditional methods for lysis often leave behind a large number of reagent residues, which makes subsequent processing on the microscale very difficult. To extract RNA and perform PCR, multiple reaction chambers are usually required.^[^ ^30^, ^31^ ^]^ Moreover, liquid can be easily lost during the transfer process between different reaction chambers.^[^ ^32^ ^]^ Traditional contact and non‐contact heating methods are used to meet the PCR thermal cycle, but these heating approaches can only serve as heat sources, making the equipment bulky. Due to the size limitations of a microchip, it would be more effective to use a multi‐functional approach that can perform thermal cycling and other processes of RNA detection simultaneously, such as cell lysis, RNA capture, RNA purification, and RNA release.

In this work, we have proposed a SAW‐based microfluidic chip to achieve reagent‐free cell lysis, RNA capture, RNA elution, PCR thermal cycling, and viral RNA detection in one chamber. The proposed chip has utilized the kinetic and thermal energy of SAW for RNA detection. The kinetic energy of SAW was used to optimize the RNA extraction process. Cell lysis was achieved by cell‐magnetic bead collisions driven by SAW‐generated whirling, where collisions disrupted the cell membrane and released RNA; RNA capture was realized by employing carboxyl‐modified magnetic beads suspended by the SAW‐induced drag force, which overcame the gravity of the magnetic beads and increased surface areas for better adsorption; RNA elution was achieved by utilizing SAW to generate high‐speed drag force, which resulted in the rapid detachment of RNA from magnetic beads. The thermal energy of SAW was used to realize thermal cycling for PCR, where three temperatures (42, 60, and 92 °C) were maintained for a desired period for reverse transcription, annealing, and denaturation, respectively, by adjusting the input power to the SAW chip. The emitting light signal was detected by a fluorescence detection module for real‐time fluorescence curve generation. The fluid control was completed automatically using preset micropumps for loading and draining reagents. Experimental results have shown that our system achieved a 350% RNA capture rate and 300% RNA elution rate compared to manual operation without the need for expensive or cumbersome devices. The PCR results from the device were comparable to those obtained from commercial qPCR instrumental detections. The proposed work shows the potential and capability of a SAW‐based lab‐on‐a‐chip device to perform the entire viral RNA detection process automatically, which provides a new option for point‐of‐care testing.

## 2 Results and Discussion

### 2.1 The Concept of the On‐Chip SAW‐Based RNA Testing

The SAW refracts into the liquid due to the transmitting speed difference as the SAW reaches the interface of substrate and water, resulting in leaky SAW with a refraction angle known as the Rayleigh angle.^[^ ^33^ ^]^ This phenomenon induces mechanical impact within the liquid. The coupling of acoustic energy into the liquid generates high‐speed flow, leading to rapid material collision and energy transfer among its components. Consequently, introducing magnetic beads into cell solutions can achieve cell lysis through fast and high‐frequency collisions induced by SAW between beads and cells. Moreover, SAW induces a drag force that facilitates bead suspension to improve the contact surface area, thus increasing the efficiency of bindings between RNA and magnetic beads. The drag force can also induce a high flow rate to improve the efficiency of RNA elution. The thermal effect can be classified into two types: thermal effect on the substrate and thermal effect in liquid.^[^ ^34^ ^]^ The former is primarily caused by mechanical and dielectric losses due to self‐heat generation in piezoelectric materials.^[^ ^35^ ^]^ The latter arises from the viscous dissipation of acoustic energy in the liquid, which includes SAW attenuation at the substrate/liquid interface and SAW‐induced acoustic streaming that heats the covering liquid along which SAW propagates.^[^ ^36^ ^]^ Therefore, reaching the required temperatures for PCR thermal cycling can be accomplished by harnessing these two forms of thermal effect. By utilizing both mechanical impact and thermal effect, together with fluidic control of the microfluidic channel, we have proposed a SAW chip to perform all necessary tasks for RNA detection.

**Figure 1:**
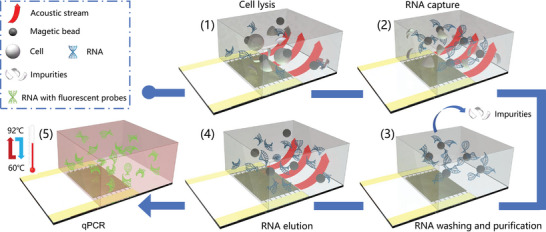
The concept of on‐chip RNA testing utilizing the mechanical and thermal effects of the SAW.

Microfluidic viral RNA detection includes five steps, as shown in **Figure** **1**: including 1) Cell lysis step, where fast‐moving magnetic beads collide with cells to disrupt the cell membrane, thus releasing RNA into the liquid; 2) RNA capture step, where SAW‐induced drag force helps RNA attach to the surface of carboxyl‐modified magnetic beads; 3) RNA wash and purification step, where unwanted impurities like cell debris and protein are separated and washed away, leaving only the magnetic beads attached with RNA; 4) RNA elution step, where RNA was separated from the magnetic beads with the help of the eluent and SAW, and an external magnetic field then captured the magnetic beads for effective RNA solution extraction; 5) qPCR step, where SAW‐induced thermal cycles were utilized to achieve temperature‐variable amplification by controlling the duty cycle of the input while generating a significant amount of fluorescent signals.

### 2.2 Design of the SAW‐Based Microfluidic Chip

**Figure 2:**
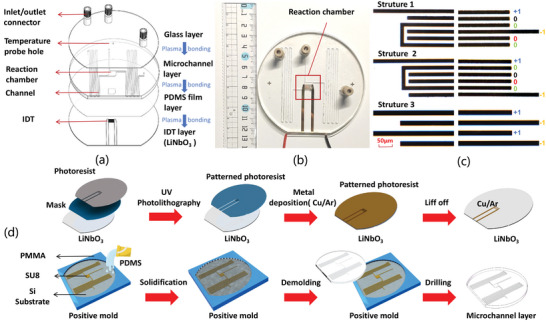
a) Three Layers of the designed microfluidic chip. b) The microfluidic chip assembly. c) Microscopic imaging of UIDT Structure 1, UIDT Structure 2, and Bidirectional IDT. d) The schematics of the fabrication process of IDT layer and microchannel layer. The detailed process can be found in Experimental Section.

The microfluidic chip consists of three layers, as shown in **Figure** **2**a,b. The top glass layer is an inlet and outlet layer that consists of four holes, including three inlet/outlet connectors for loading and discharging liquid, and one temperature probe port. The second layer is made of Polydimethylsiloxane (PDMS), which consists of a reaction chamber that holds various reactions and a loading channel that pre‐stores various reagents. The third layer is made of PDMS film, which seals the microchannel in the second layer. The 100‐µm‐thick PDMS film has a hollowed‐out middle section, which serves as the reaction chamber and allows the SAW energy to penetrate it effectively. The bottom layer is a lithium niobate (LiNbO_3_) substrate that is coated with an IDT and responsible for generating SAW for reaction processes. The different layers are bonded together through plasma treatment conducted on the PDMS surface at 85 W for 17 s. The specific desgin parameters can be found in Experimental Section.

We have designed six types of IDTs using 128°Y‐cut LiNbO_3_ with three design variables, including the central frequency, the number of finger pairs, and the layout of fingers. The baseline frequency was chosen to be 32.4 MHz, which is the harmonic frequency of the ϕ1µm silica magnetic beads used in our experiment. We have designated the electrode connected to the positive bus bars as “+1”, the electrode connected to the negative bus bars as “‐1”, and the floating electrode as “0”. Three different layouts have been created and displayed in Figure 2c. The first layout, Structure 1, is a uni‐directional IDT (UIDT) consisting of two floating electrodes placed between the fixed electrodes.^[^ ^27^ ^]^ This layout is identified as having a “+100‐100” layout. The second layout, Structure 2, is similar to Structure 1, but with three floating electrodes between the fixed electrodes.^[^ ^37^ ^]^ It is identified as having a “+10000‐1” layout. Finally, Structure 3 is a bidirectional IDT without floating electrodes and has a “+1‐1+1‐1” layout. The parameters of six different SAW chips are illustrated in **Table** **1**. The harmonic frequency simulation of the magnetic bead is described in Figure S1 (Supporting Information); The actual central frequency of the IDT designed with the baseline frequency is 32.4 MHz as described in Figure S2 (Supporting Information).

**Table 1:** Parameters of Six Different SAW Chips.

IDT No.	Layout	Finger width (µm)	Frequency (Mhz)	Pairs
IDT 1	Structure 1	12	22	40
IDT 2	Structure 1	10.2	32.4	40
IDT 3	Structure 1	10.2	32.4	80
IDT 4	Structure 1	5.4	61.4	40
IDT 5	Structure 2	10.2	32.4	40
IDT 6	Structure 3	10.2	32.4	40

### 2.3 The Evaluation of the SAW‐Based Cell Lysis

**Figure 3:**
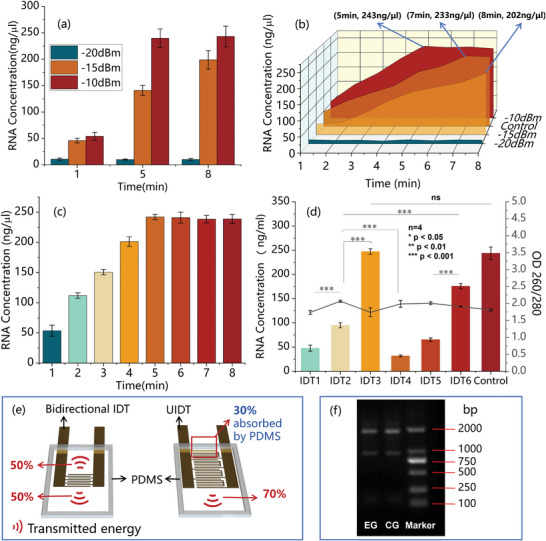
a–c) The evaluation of SAW chip input power and lysis time in cell lysis by cell‐microparticle collisions. a) The RNA concentration obtained with three input powers and three reaction periods (1, 5, and 8 min). b) The RNA concentration trend with different input parameters. c) The concentration of RNA extraction at different time points from 1–8 min with an input power of ‐10 dBm. d) RNA concentration and OD 260/280 of different IDTs. With an input power of ‐10 dBm. e) Energy transmission of two types of IDTs: The energy of bidirectional IDT was completely released into the reaction chamber. The energy generated at the rear end of the UIDT(Structure 1 and Structure 2) was absorbed by PDMS. f) Gel electrophoresis: Experimental group (EG): RNA extracted by IDT 3; Control group (CG): RNA extracted by manual operation.

Before evaluating the effectiveness of six IDTs in cell lysis, we have determined the input power and lysis time of the SAW for cell lysis utilizing cell‐microparticle collisions. As a control group, we have used EpiQuik Magbeads Quick RNA Isolation kit (p‐9106‐050, EpiQuik, USA), 95 µl Hela cells (2.5 × 10^5^ cellsml^‐1^), and 5 µl magnetic beads (7 × 10^9^ beadsml^‐1^) for lysis as the control group. All steps except lysis were manually performed to ensure the control of variables, Lysis was performed in a reaction chamber (Figure 2b). The concentration of total RNA extraction was used as an indicator for assessing lysis efficiency. IDT three was utilized to lyse cells with ‐20 dBm, ‐15, and ‐10 dBm with durations of 1, 5, and 8 min, respectively. When using an input power of ‐10 dBm, higher lysis efficiency was achieved compared to ‐15 dBm and ‐20 dBm, as shown in **Figure** **3**a. We further compared the three input powers' lysis efficiencies with those of the control group at different time points. In the control group, RNA concentration increased over time, reaching its peak at around 7 min, as illustrated in Figure 3b. With an input power of ‐20 dBm, RNA concentrations were extremely low, indicating minimal cell lysis occurred. When the input power was increased to ‐15 dBm, the RNA concentration increased over time for 8 min, but it did not achieved the desired RNA concentration. However, the desired RNA concentration was reached within 5 min when an input power of ‐10 dBm was achieved (Figure 3c). Further increase in power would result in a rapid temperature rise. Therefore, ‐10 dBm and 5 min were identified as the optimal parameters for subsequent cell lysis using magnetic beads collisions.

The following results were obtained: 1) The RNA concentration obtained from IDT 2 was found to be 100% and 200% higher than that from IDT 1 and IDT 4. This indicates that the IDT designed with the first harmonic frequency of magnetic beads has obtained significantly higher RNA concentrations. 2) RNA concentration obtained from IDT 6 was 73% and 170% higher than that from IDT 2 and IDT 5. Meanwhile, RNA concentration obtained from IDT 2 was 58% higher than that from IDT 5. The bidirectional IDT structure demonstrated superior cell lysis compared to the UIDT structure. Structure 1 achieved better cell lysis than Structure 2. 3) RNA concentration obtained from IDT 3 was 135% higher than that from IDT 2. This indicates that IDT designed with a higher number of finger pairs have obtained significantly higher RNA concentrations. The evaluation results obtained are illustrated in Figure 3d. This outcome aligns with expectations: 1) Resonance‐induced vibration amplifies the vibrational amplitude through energy accumulation, resulting in magnitudes several times larger than those achieved by continuous non‐resonant excitation.^[^ ^38^, ^39^ ^]^ 2) Bidirectional IDT generated two‐directional acoustic streaming with nearly total output energy when positioned at the center of the reaction chamber (Figure 3e). In contrast, energy leakage from the rear end of UIDT (approximately 30%) was absorbed by a PDMS barrier (Figure 3e),^[^ ^40^ ^]^ resulting in reduced mechanical stimulation to the cell‐bead solution and, consequently, lower effectiveness compared to the bidirectional IDT. 3) Increasing the number of finger pairs amplifies SAW amplitude, which enhances cell lysis efficiency.

Therefore, four guidelines can be concluded for selecting IDTs for cell lysis: 1) Selecting the harmonic frequency of the magnetic bead as the central frequency of the IDT to achieve effective cell lysis by energy accumulation during the cell‐microparticle collision; 2) Increasing the number of IDT finger pairs to enhance cell lysis efficiency by amplifying SAW amplitude; 3) Increasing the input power to improve RNA extraction concentration up to the full adsorption capacity of magnetic beads; 4) Separating the interdigital transducer from the barrier of the reaction channel as much as possible to minimize the acoustic energy adsorption by the barrier, thus improving cell lysis outcome.

Among all tested IDTs with an input power of ‐10 dBm, IDT three achieved RNA concentrations similar to manual operation. The OD values of all IDTs fell within a reasonable range. The integrity of RNA obtained through IDT three was assessed using gel electrophoresis analysis, revealing no significant (ns) differences between their results and that of the control group (Figure 3f). With all IDTs on hand, IDT three achieved the best results among all. The gel electrophoresis analysis of other IDTs can be found in Figure S5 (Supporting Information). Considering that the extraction effect achieved by IDT 3 is sufficient to meet the requirements, IDT three was selected for our microfluidic platform.

**Figure 4:**
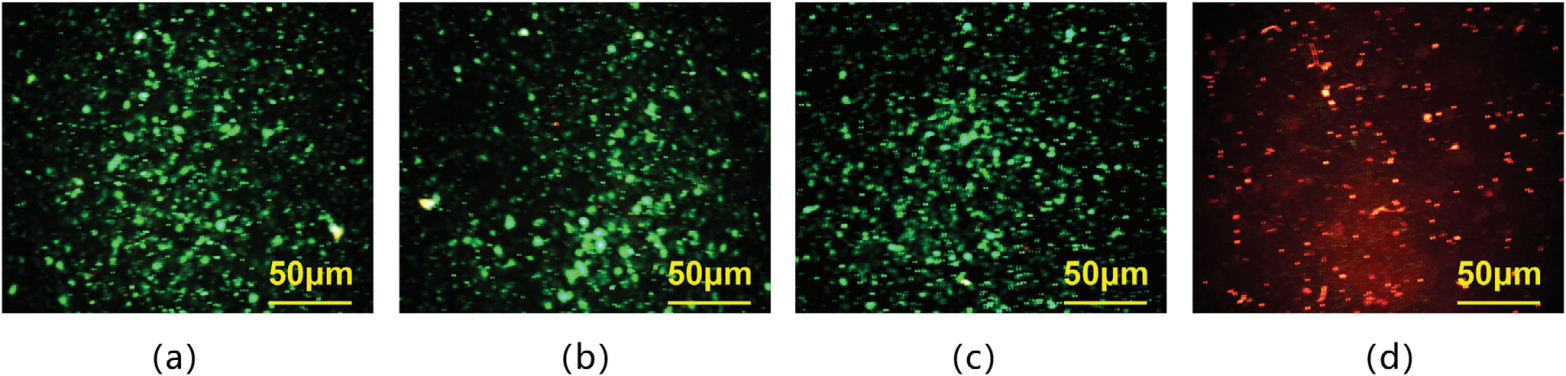
Fluorescence microscopy with AOPI staining was used to observe the extent of Hela cell lysis by SAW in the presence or absence of magnetic beads. a) Cells showed fluorescent green without SAW or magnetic beads. b) Cells remained fluorescent green with SAW but without magnetic beads. c) Cells showed fluorescent green without SAW but with magnetic beads. d) Cells turned fluorescent red with SAW and magnetic beads.

To confirm that cell lysis was achieved through magnetic bead collisions rather than lysis by SAW alone, We have conducted a control acridine orange and propidium iodide (AO‐PI) staining experiment, using 100 µl Hela cell sample as the control group and 100 µl cell‐magnetic beads mixture (95 µl cells, 5 µl magnetic beads) as the experimental group (as shown in **Figure** **4**). AO stains both live and dead cells and displays green light under excitation light at a wavelength of 485nm, whereas PI stains only the dead cells and displays red light under excitation light at 485nm. Both groups with or without magnetic beads showed fluorescent green initially, as shown in Figure 4a,c. When we set the SAW input power to ‐10 dBm and ran it for 5 min, many cells remained green in the group without magnetic beads, indicating minimal influence on cell lysis by SAW alone, as shown in Figure 4b. However, most cells showed fluorescent red in the group with magnetic beads, indicating cytomembrane rupture occurred, as shown in Figure 4d.

### 2.4 The Evaluation of the SAW‐Based RNA Capture

RNA capture was achieved by specific adsorption of carboxyl groups on the surface of magnetic beads. To capture nucleic acid, negatively charged phosphoryl groups interact with carboxyl groups on magnetic beads, via Na+ ion bridges in the presence of PEG and NaCl. After removing the reaction buffer, adding aqueous molecules hydrates the nucleic acid, breaking the ionic interaction, and purifying the nucleic acid adsorbed on the magnetic beads. At the RNA capture stage, due to the force of gravity, most magnetic beads in the solution settled at the bottom of the reaction chamber, resulting in a reduced surface area available for RNA binding. Without external forces, buoyancy alone was insufficient to counteract gravity and suspend the magnetic beads. Therefore, we introduced SAW to induce a drag force facilitating bead suspension. Precise control of input power applied to the IDT is crucial. Inadequate power leads to low drag forces. Excessive input power increases the flow rate and diminishes efficient RNA capture. Thus, an optimal input power should generate a sufficient flow rate and sustain bead suspension, thus maximizing contact surface areas for RNA binding.

**Figure 5:**
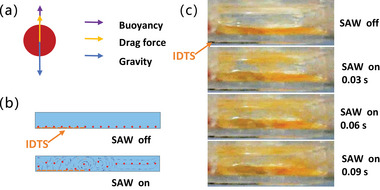
a) Force analysis of a magmatic bead. b) Schematic drawing of simulation of magnetic beads in the reaction chamber with the SAW on and off. c) Image of magnetic beads in a rectangular chamber with the SAW on and off. Simulation results can be found in Figure S6 (Supporting Information).

A magnetic bead experiences three forces in the solution, the drag force, the gravity force, and the buoyancy force, as illustrated in **Figure** **5**a. The drag force, *F*_*D*_ can be estimated^[^ ^27^ ^]^ as

(1) $$F_{D}=\frac{1}{2}\rho \upsilon^{2}C_{D}A•\cos\left(22^{\circ }\right)$$

where ρ, υ, *C*_*D*_, and *A* are the density of the liquid, velocity of fluidic flow, drag coefficient, and cross‐section area of a particle, respectively. That drag coefficient was estimated to be 0.45 as a high‐Reynolds sphere in water.^[^ ^27^, ^41^ ^]^ The buoyance can be calculated as follows:

(2) $$F_{B}=\frac{4}{3}\pi r^{3}\rho g$$

where *r* and *g* are particle radius and gravitational acceleration. The gravity is estimated as:

(3) $$F_{g}=\frac{4}{3}\pi r^{3}\rho_{p}g$$

where ρ_*p*_ is the density of the particle. Therefore, the optimum input parameter of the IDT should generate right amount of *F*_*D*_ to overcome the *F*_*g*_ together with *F*_*B*_.

We have simulated the movement of magnetic beads in the reaction chamber. The simulation result has shown that the magnetic beads were suspended as the SAW‐induced drag forces effectively counteracted gravity, as shown in Figure 5b,c. To observe the phenomenon, we have also conducted a experiment on SAW‐driven magnetic beads in a device with a similar structure. We placed 50 µl of magnetic beads suspension (7 × 10^9^ beadsml^‐1^) in front of the IDT with an input power of ‐15 dBm for analysis and observation. The magnetic beads settled on the substrate before introducing acoustic streaming, as shown in Figure 5b,c. Upon activation of the SAW, magnetic beads were propelled along the Rayleigh‐angle direction and eventually suspended within the vortex generated by the acoustic stream in the droplet, as shown in Figure 5b,c.

**Figure 6:**
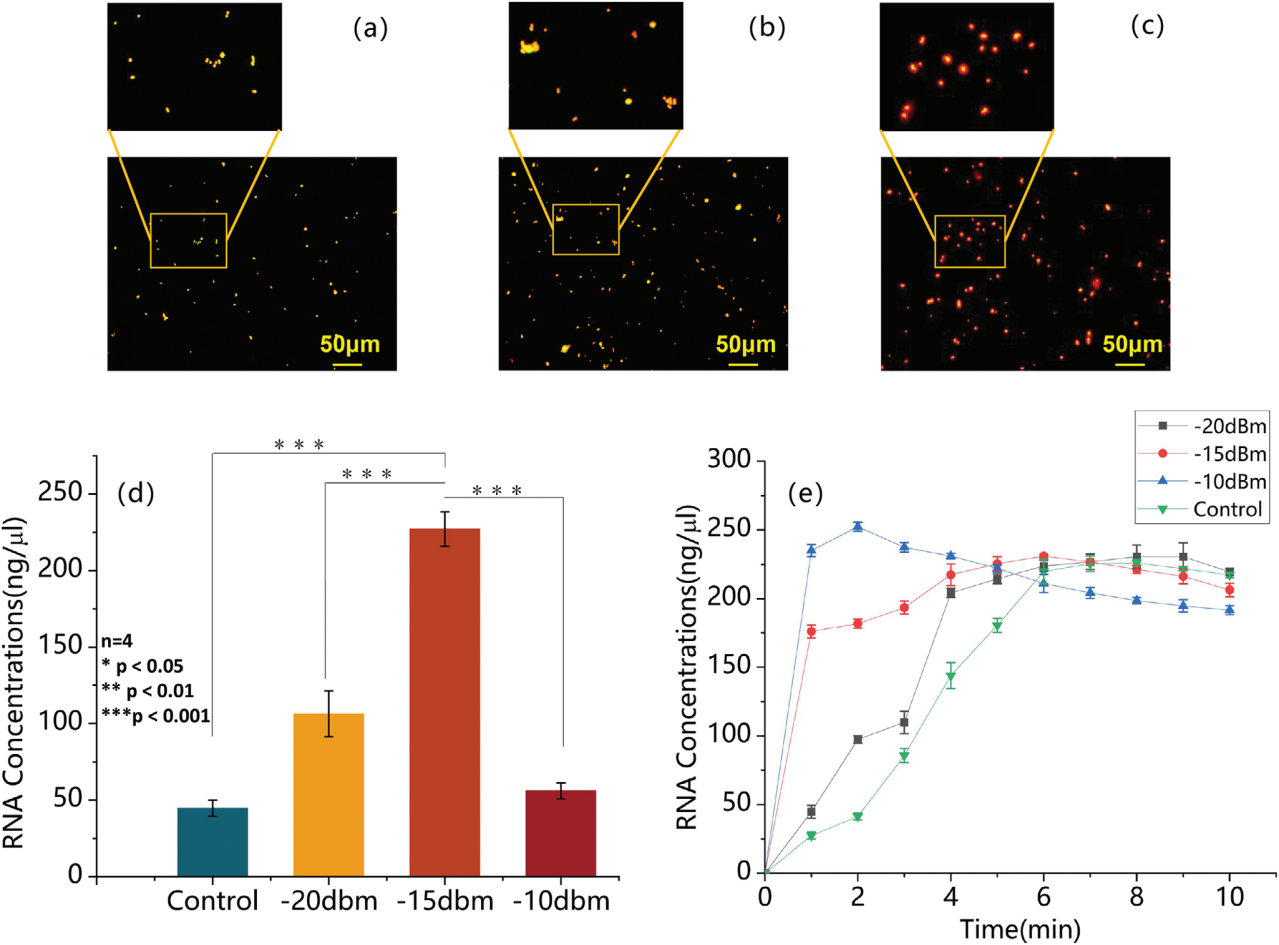
a–c) Fluorescent staining of RNA and magnetic beads. RNA and magnetic beads show red and yellow fluorescence at 490 nm excitation wavelength, respectively. a) Yellow fluorescent magnetic beads without RNA adsorption under a 490 nm excitation wavelength. b) The fluorescent staining of a combination process of RNA and the magnetic beads without SAW. c) The fluorescent staining of a combination process of RNA and the magnetic beads with ‐15 dBm input SAW. d) RNA concentration captured by magnetic beads with different input powers. e) RNA concentration versus elution time with different input powers.

We have stained the RNA and captured it with fluorescent‐labeled magnetic beads. A confocal microscope (TSC‐sp2, Leica, Germany) was used to observe the adsorption of RNA (30 µl, 247ngµl^‐1^) by magnetic beads (1 µl, 7 × 10^9^ beadsml^‐1^) with different SAW input powers. The magnetic beads and RNA appeared yellow and red under 490nm wavelength excitation light (the fluorescence of the magnetic beads is shown in **Figure** **6**a). The RNA capture period was set to 5 min in our experiment. The experimental results have shown only a small amount of RNA adsorption around the magnetic beads without SAW, as shown in Figure 6b. However, when introducing SAW with ‐15 dBm, there was a large amount of RNA around the magnetic beads, as shown in Figure 6c, which indicated that the suspension of magnetic beads caused by SAW could improve the capture efficiency of magnetic beads for RNA.

We have further analyzed the RNA capture efficiency with different input powers. The concentration of total RNA extraction was used as an indicator for assessing capture efficiency. The application of SAW has led to a noticeable enhancement in the RNA adsorption efficiency, as illustrated in Figure 6d. Notably, at an input power of ‐20 dBm, there was a significant increase in the RNA concentration compared to the Control. The RNA concentration was further improved to 227ngµl^‐1^ when the input power was set to ‐15 dBm, which achieved 350% RNA capture rate compared with manual operation. However, a substantial decline in concentration was observed when the input power was set to ‐10 dBm, resulting in a substantial decline in concentration. These observations have aligned with our previous description wherein low input power induced gentle acoustic streaming that facilitated magnetic bead suspension and RNA capture. However, higher input powers hindered the combination process between RNA and magnetic beads due to increased drag forces, making it harder for RNA to bind to the magnetic beads. Moreover, excessive input powers caused rapid temperature elevation and evaporation. Therefore, we have selected an input power of ‐15 dBm for optimal RNA capture.

### 2.5 The Evaluation of the SAW‐Based RNA Elution

Increasing the SAW input power can improve RNA eluting efficiency by inducing a high drag force due to a high flow rate. The drag force in fast fluidic flow can help detach the RNA from the magnetic beads. The evaluation criterion for elution was the time required to achieve the desired RNA concentration. Before switching on the SAW, 50 µl elution buffer was filled into the reaction chamber. Three input powers were tested, including ‐20, ‐15, and ‐10 dBm. It took 8 min for the control group to reach the desired concentration, as shown in Figure 6e. The elution period for reaching the same concentration was shortened to 7 min when the SAW was turned on with an input power of ‐20 dBm. As the input power increased, the time required to reach the desired RNA concentration was also reduced. Thorough elution took 5 and 1 min as the input power was adjusted to ‐15 and ‐10 dBm, respectively. Experimental results have shown that with an input power of ‐10 dBm, elution took 300% less time compared with the control group. It took 2 min to reach the desired concentration (≈258 ngµl^‐1^). RNA concentration showed a downward trend after reaching the peak value, presumed to be a certain degree of RNA degradation. Further increasing the input power raised the temperature rapidly and led to fast evaporation; thus, we chose ‐10 dBm or lower input power for RNA elution and 258 ngµl^‐1^ as the RNA template's concentration for qPCR.

### 2.6 The Evaluation of SAW‐Induced Thermal Cycles for PCR

When a SAW propagating on the substrate surface encounters liquid, the SAW radiates acoustic energy into the liquid due to the sound speed mismatch between the substrate and the liquid, which initiates a pressure wave and drives a steady‐state flow called acoustic flow. At the same time, the dissipation of acoustic energy in the fluid will have a thermal effect.^[^ ^42^, ^43^ ^]^ Utilizing this thermal property of SAW, one can perform a thermal cycle with different power inputs to the IDT.

**Figure 7:**
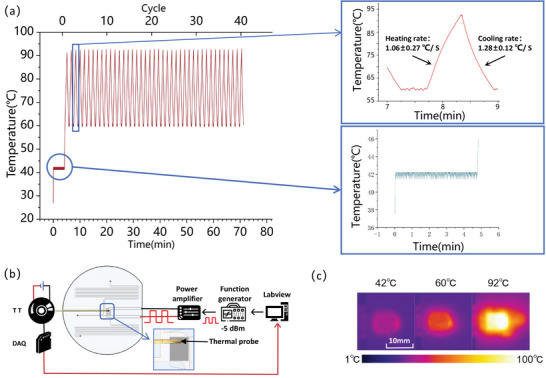
a) PCR circulation temperature curve based on SAW. b) Schematics of temperature control, and the detailed process can be found in the Experimental Section. c) Infrared thermal imaging of SAW heating in the reaction chamber.

We have controlled and monitored the temperature fluctuations of the fluid induced by SAW within the reaction chamber. In our experiment, the reaction chamber was first filled with 60 µl PCR premixed solution. According to the PCT kit manual (AG11701, AG, China), reverse transcription, denaturation, and annealing require a temperature of 42, 92, and 60 °C, respectively, and the detailed temperature and period for each step are described as follows: 1) reaction at 42 °C for 5 min; 2) reaction at 92 °C for 30 s; (3) reaction at 92 °C for 5 s and 60 °C for 30 s for 40 thermal cycles. We have evaluated the thermal effects of the selected IDT for various target temperatures. When setting the input power to ‐5 dBm, it took 20 s to heat the reaction chamber from room temperature (27 °C) to 42 °C, 30 s to increase the temperature from 42 to 60 °C, and 30 s to raise the temperature from 60 to 92 °C. The time of one thermal cycle was approximately 100 s. The SAW chip can achieve forty 92–60 °C thermal cycles within 65 min, as illustrated in **Figure** **7**a. Temperature control was achieved by changing the duty cycle of the SAW input power. Cooling process occurs naturally through heat dissipation from chamber boundary. It begins immediately after power is cut off, as shown in Figure 7b. The temperature probe was placed in the upper center of the reaction chamber. Throughout the reaction, the chamber was filled with a reagent that immediately transferred heat, resulting in a nearly uniform temperature in the chamber. All temperatures could be controlled accurately with an average error of ±0.5 °C, as shown in Figure 7c. As a result, the temperature recorded by the probe reflected the temperature of the chamber. In brief, we have accomplished thermal cycling on the SAW‐based RNA detection device, and its efficiency has fulfilled the requirements of PCR.

### 2.7 Workflow of the Automatic Fluidic Control Process for RNA Detecting

**Figure 8:**
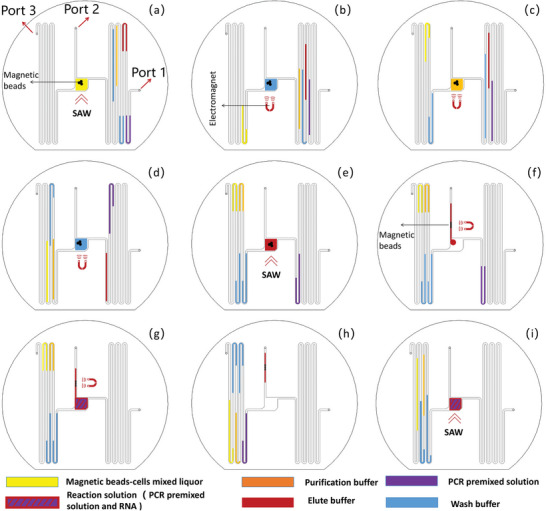
Workflow of the automatic fluidic control process. a) Cell lysis by SAW‐induced magnetic bead collisions. b) Washing off the impurities with the washing solution. c) Washing away DNA and proteins with a purified solution. d) Repeating the washing process for a second time. e) Filling eluate into the reaction chamber. f) Separating magnetic beads with a magnetic field. g) Mixing RNA with the PCR premix. h) Blotting off excess RNA samples. i) SAW‐induced PCR thermal cycling. The more information can be found in Video S1 (Supporting Information).

**Figure 9:**
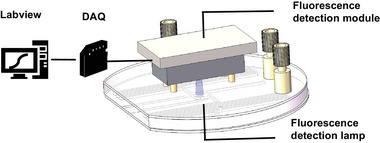
Fluorescence sensor positioned above microfluidic chip to collect fluorescence signal.

We have implemented a programmed flow control using motorized microinjectors and valves to automate RNA detection. The workflow of the automatic fluidic control process is illustrated in **Figure** **8**. All ports are connected with microinjectors via controllable valves for liquid loading and disposal. The workflow includes five steps: 1) Prior to initiating the reaction, specific volumes (100 µl of washing solution, 80 µl of purification solution, 100 µl of washing solution, 50 µl of eluate, and 59 µl of PCR premix) were injected into the microchannel through port one with interposed air gaps (each containing 100 µl) to separate different reagents. A preload of 5 µl magnetic beads and 95 µl cells was introduced into the reaction chamber through port 2 (100 µl) to prevent cross‐contamination between liquids during the injection process, as depicted in Figure 8a. The SAW was activated for cell lysis by inducing collisions between magnetic beads and cells. Subsequently, incubation was carried out for 5 min; 2) Stabilize the magnetic beads in a fixed position by applying an external magnetic field, and then allow mixing between the washing solution and the cell‐magnetic bead mixture, which is then passed through the reaction chamber to remove unwanted impurities and cell debris, as depicted in Figure 8b; 3) Filling the reaction chamber with purification solution for 5 min before discharging it completely to achieve thorough purification, as depicted in Figure 8c; 4) Filling the reaction chamber with washing solution for 5 min, followed by complete discharge, as depicted in Figure 8d; 5) Deactivate the external magnet and turn on the SAW after loading eluate into the reaction chamber. This allowed RNA release to occur efficiently, obtaining high‐concentration RNA samples, as depicted in Figure 8e; 6) Removing the excessive RNA solution from port two, leaving only 1 µl for subsequent PCR amplification. Magnetic beads were immobilized in the microchannel near port two using an external magnetic field, as depicted in Figure 8f; 7) Mix the PCR premix remaining RNA solution in the reaction chamber, as depicted in Figure 8g; 8) Port one and three were opened while port two was closed, allowing the sample PCR reaction solution to be aspirated into the channel; this step aimed to separate the PCR reaction solution from the residual eluate, as depicted in Figure 8h; 9) After closing port one and opening ports two and three, the PCR reaction solution was drawn into the reaction chamber for thermal cycling preparation, as depicted in Figure 8i. Experimental results demonstrate that the entire virus detection process, from “sample to answer”, takes only about 90 min, whereas traditional methods often require up to 4–8 h to process the samples and an additional 1–3 days to report results.^[^ ^44^ ^]^ We have also used an electronic microbalance to measure the amount of liquid lost after the reaction and found that only 1.3% of the liquid (60.79 mg) was lost compared to the initial amount (61.61 mg). Microscopic observations revealed that the remaining liquid was in the port three channel, which is used to drain waste liquid after the reaction. Therefore, the residual reagent doesn't have any effect on the PCR reaction. Detailed liquid control during the experiment can be found in Figure S4 (Supporting Information).

### 2.8 The Evaluation of the SAW‐Based RNA Detection

We have evaluated the performance of the proposed SAW‐based device for complete RNA detection. The entire RNA detection process includes RNA extraction and PCR. To assess the accuracy of results, we have utilized the current real‐time quantitative PCR (qPCR) instrument (480II, Roche, Switzerland) as a benchmark. We have used three different pseudovirus, pCDH‐CMV‐MCS‐EF1‐copGFP‐T2A‐Puro (T2A, RUYAO, China), Recombinant Protein (GIPC1, RUYAO, China), and CKLF like MARVEL transmembrane domain containing three Gene (CMTM3, RUYAO, China), as the source of infection. We have selected Hela cells as infected samples, considering the higher infection efficiency of this lentivirus on them. The infected cells were used as positive test samples, and the uninfected cells were used as negative test samples. Infected cells carry the copepod green fluorescent protein (copGFP) gene used as a target gene (the process of infection, primer sequences, and probe sequences can be found in the Experimental Section). Fluorescence signals from target genes were recorded and evaluated in real‐time in SAW‐based PCR. Fluorescence emitted from the sample was detected by a fluorescence sensor positioned above the reaction chamber during thermal cycling, as depicted in **Figure** **9**.

**Figure 10:**
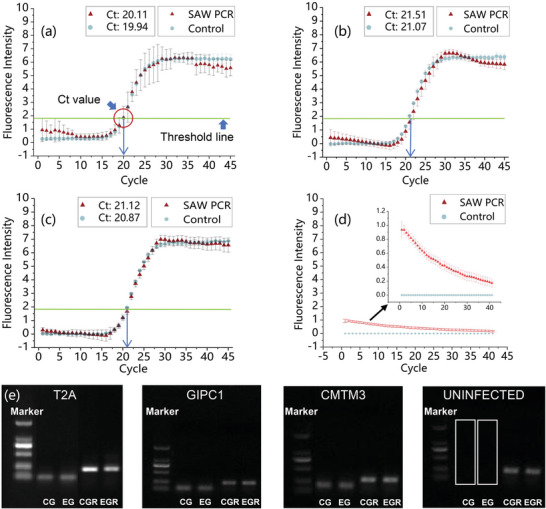
a) PCR amplification result of T2A infected sample. The mean squared error of the SAW PCR group (MSE‐S) was 0.63, the mean squared error of the control (MSE‐C) was 0.28. b) PCR amplification result of GIPC1 infected sample. MSE‐S:0.035; MSE‐C:0.011. c) PCR amplification result of CMTM3 infected sample. MSE‐S:0.031; MSE‐C:0.007. d) PCR amplification result of the negative test sample. MSE‐S: 0.063; MSE‐C: 7.7x10^−6^. e) Gel electrophoresis analysis of the amplicons. The original full image of gel electrophoresis analysis can be found in Figure S7 (Supporting Information).

The amplification curve of the positive test sample reached its exponential phase and plateaued at the 14th and 20th thermal cycles, respectively. The sigmoidal shape of the fluorescence curve has indicated a successful PCR amplification, as illustrated in **Figure** **10**a–c. There was no significant difference between the SAW‐based PCR and the control. The modest decline in the fluorescence curve during the baseline and plateau phases was attributed to fluorescence quenching and acquisition errors. Conversely, the amplification curve of the negative test sample remained flat with a slight downward trend throughout all 40 thermal cycles due to fluorescence quenching, as illustrated in Figure 10d. The fluorescence curves obtained from the SAW‐based device were not smooth and straight like those from the control. This was due to the fact that the data from the SAW‐based device was raw and unprocessed. Conversely, the data from the control had undergone smoothing in the qPCR instrument, which resulted in a smaller mean squared error (MSE).

We have also performed gel electrophoresis, where the SAW‐based approach yielded comparable outcomes to conventional PCR equipment, thereby validating the amplification and specificity, as depicted in Figure 10e. The infected samples in the control group (CG) were obtained from the commercial qPCR instrument. The infected samples in the experimental group (EG) were obtained from SAW‐based device. The reference gene samples for the control group (CGR) were obtained from the commercial qPCR instrument. The reference gene samples for the experimental group (EGR) were obtained from SAW‐based device. These experimental results have further verified that the proposed device has yielded comparable outcomes to those achieved through commercial qPCR instrumental detection.

## 3 Conclusion

In this study, we have designed an on‐chip RNA detecting device based on SAW, achieving programable controlled in situ cytolysis‐RNA capture‐RNA release‐RNA amplification‐PCR in one chamber. We have utilized the proposed device to lyse cells and release RNA by SAW‐induced cell‐microparticle collision, capture RNA in situ through SAW‐suspended magnetic beads, and elute RNA with SAW‐generated high drag force generated by SAWs. Finally, we used the thermal effect of SAWs to perform PCR thermal cycling, during which the amplified fluorescent signals were acquired and analyzed. The results obtained from our proposed device were comparable to the benchmark result from the commercially available instrument, demonstrating the potential of SAW‐based lab‐on‐a‐chip devices for point‐of‐care testing. This study has addressed various issues related to SAW‐based all‐in‐one assays. These include dynamic analysis for cell lysis and RNA extraction in a sealed chamber, precise temperature control induced by SAW for PCR thermal cycling, and logic control for a fully automated virus detection. The device is designed to be reusable after cleaning and sterilization, making it more cost‐effective. It also offers the flexibility of being pre‐filled with various reagents using capsule arrays and pre‐filled auto‐injectors. Additionally, it can be developed as a portable and all‐in‐one machine for automated virus detection on microfluidic chips, making it potentially useful for other biochemical assays like immunoassays, proteomics, and enzyme activity assays. Further work will focus on designing multiple reaction chambers and microchannels for parallel testing, optimizing the heating and cooling control for faster thermal cycling, improving the limit of detection for better sensitivity, and miniaturizing the proposed device for portability and affordability.

## 4 Experimental Section

### Device Design and Fabrication

A layer of photoresist (AR‐N 4340, AR, Germany) was coated on the surface of 128°Y‐cut LiNbO_3_ (Thickness of 1 mm; Diameter of 100 mm) with a speed of 1000 rpm for 6s and 5500 rpm for 20s. Then, the wafer was patterned with a UV light source under a predesigned mask and developed in the photoresist developer. Then, a double metal layer (Cr/Au, 5 nm/80 nm) was deposited on the wafer through an e‐beam evaporator. At last, the IDTs were formed by a lift‐off process, as shown in Figure 2d The PDMS micro channels were formed by a standard photolithography process using SU8 as the template on a separated Si substrate. The internal cross‐sectional dimension of the channel was 15 µm × 15 µm, and the reaction chamber dimension was about ≈8 mm × 8 mm × 1 mm. The length of the PDMS channel was 400, 30, and 600 mm respectively, and a 1000 µm hole was made by a medical biopsy puncture tool in one end of the PDMS channel for fluid pipe connection. Once the PDMS channels were ready, they were bonded on the LN substrate with the LN substrate treated by oxygen plasma. The above devices were immersed in a 10% ribonuclease inhibitor (AG21302, AG, China) for 10 min before the experiments to prevent the influence of ribonuclease. The PDMS channel was washed with alcohol before the reaction to prevent contamination. The whole experimental setup is shown in Figure S3 (Supporting Information).

### System Setup

The SAW device was connected with an arbitrary/function waveform generator (33250A, Agilent, USA) in conjunction with an amplifier (LZY‐22+ 0.1‐200 MHz, Mini Circuits, USA) and a 10 A and ±24 V DC power supply. The arbitrary/function waveform generator was connected with a computer. The power, frequency, and duty cycle of the SAW were controlled using LABVIEW (2021(X64), NI, USA).

### Fluorescence Signal Acquisition

Fluorescence signal acquisition was detected through the fluorescence detection module (MNS‐MFD‐470, Monaisi, China). A data acquisition (DAQ) card was connected with one end of the fluorescence detection module, and the fluorescence signal was visualized by LABVIEW.

### Temperature Control

The temperature detection was accomplished by using a temperature transmitter. A temperature probe was inserted into the reaction chamber to measure and collect temperature data precisely. Real‐time temperature data was transmitted to the computer through DAQ and then controlled and adjusted by LABVIEW using PID control to regulate the duty cycle of IDT output power, thereby achieving precise temperature stability control. (Figure 7c).

### Microfluidic Control

Microfluidic control was achieved using microvalves (P20NC‐12‐01, Eldon James, USA), electromagnets (YR‐P10/25, CNYRIL, China), and motorized microinjector (SPC, DK Infusetek, China). Microvalves were used to clamp tubes to stop the liquid flow and provide desired flow direction; Electromagnets were used to stabilize the magnetic beads during washing and elution; Microinjectors were used to inject liquid into the microchannel. The microvalve and the electromagnet were connected with a 5V electromagnetic relay and an IO board. LABVIEW controlled this switching cycle. The microinjector was equipped with a microsyringe, which was connected to the inlet and outlet with a ϕ1 mm silicone tube, as shown in Figure S3 (Supporting Information).

### Cell Culture and Lentiviral Transfection

Hela cells were cultured in medium (DMEM), supplemented with 5% elitegro‐adv (SuperCulture, New Zealand). Before lentivirus transfection, Hela cells in good condition were inoculated into 12‐well plates at a cell density of 1x10^5^/ml, and incubated at 37 °C overnight. Then, the original medium of the cells was removed, and 500 volume of culture medium (fresh medium mixed with 50% virus stock) was added. Infection was performed at 37 °C for 4 h, and the culture medium was replenished to 1ml after 4 h. On the third day after infection, a fresh complete medium was replaced, and 3 µl Puromycin (1 µgµl^‐1^) was added on the fifth day after infection. Subsequent subcultures were performed every two days, the complete medium was replaced, and 3 µl Puromycin (1 µgµl^‐1^) was added.

### Probe and Primer Sequence

Probe sequence (5'‐FAM‐TCTTCCGCCTGCCACACTTA‐BHQ1‐3'); upstream primer sequence (5'‐ACGCTGTATGTAGTTCTC‐3'); downstream primer sequence (5'‐CAAGCA‐CCATGTCATATTG‐3').

### Significant Difference Analysis

Analysis of significant differences was done by data analysis software (2021(x64), OriginLab, USA) using monofactor analysis measures ANOVA.

## Conflict of Interest

The authors declare no conflict of interest.

## Supporting information

Supporting Information

Supplemental Video 1

## Data Availability

The data that support the findings of this study are available from the corresponding author upon reasonable request.
