# Supplementary material for: A SAW‐Based Programmable Controlled RNA Detecting Device: Rapid In Situ Cytolysis‐RNA Capture‐RNA Release‐PCR in One Mini Chamber
Source: Adv Sci (Weinh). 2024 May 21;11(29):2309744. doi: 10.1002/advs.202309744 (PMC11304306; doi:10.1002/advs.202309744)
Supplement: Supplementary file 1 — Supporting Information [file ADVS-11-2309744-s001.pdf]

ADVANCED SCIENCE
Open Access

# Supporting Information

for *Adv. Sci.*, DOI 10.1002/advs.202309744

A SAW-Based Programmable Controlled RNA Detecting Device: Rapid In Situ Cytolysis-RNA Capture-RNA Release-PCR in One Mini Chamber

*Yupeng Yang, Zenan Wang*, Hetao Xie, Ying Hu and Hong Liu**

WILEY-VCH

ADVANCED SCIENCE Open Access

*Supporting Information*

# A SAW-Based Programmable Controlled RNA Detecting Device: Rapid In-Situ Cytolysis-RNA Capture-RNA Release-PCR in One Mini Chamber

Yupeng Yang§, Zenan Wang§*, Hetao Xie, Ying Hu, Hong Liu*

§ Yupeng Yang and Zenan Wang contributed equally.

*Supporting Information*

# A SAW-Based Programmable Controlled RNA Detecting Device: Rapid In-Situ Cytolysis-RNA Capture-RNA Release-PCR in One Mini Chamber

Yupeng Yang§, Zenan Wang§*, Hetao Xie, Ying Hu, Hong Liu*

Y. Yang, Z. Wang, Y. Hu
Shenzhen Institute of Advanced Technology, Chinese Academy of Sciences 518000
Email Address:zn.wang1@siat.ac.cn
Y. Yang, H. Xie, H. Liu
University of Jinan, Jinan 250022, P. R. China
Email Address:hongliu@sdu.edu.cn

# Supplementary figures

The magnetic bead was assigned Young's modulus and shear modulus values of 2.7618e+9 pa and 3.1197e+10 pa, respectively. Simulation results indicated that the magnetic bead experienced the most significant deformation at the first harmonic frequency of 32.4MHz. Therefore, we designed the IDT to operate at the first harmonic frequency of the magnetic bead.

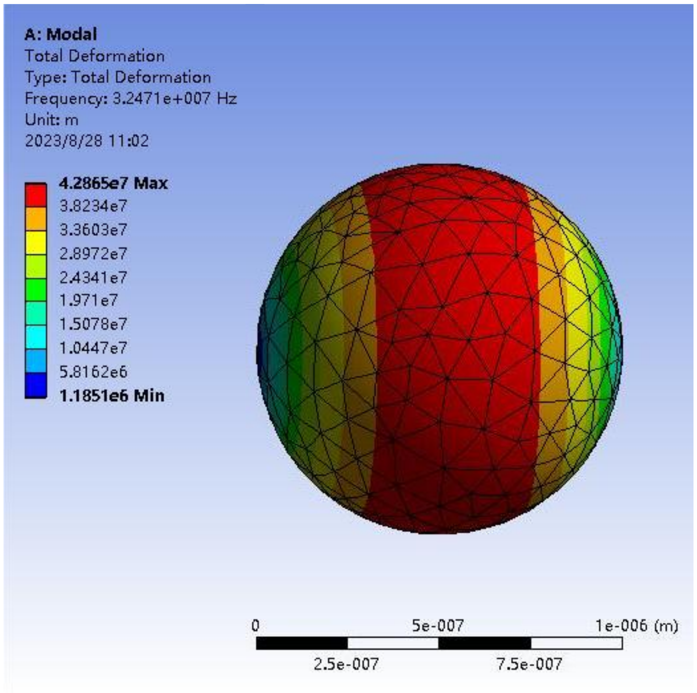


| Properties | |
|---|---|
| Volume | 5.236e-19 m³ |
| Mass | 1.3614e-15 kg |
| Centroid X | -1.6381e-24 m |
| Centroid Y | 1.0798e-23 m |
| Centroid Z | -1.2871e-24 m |
| Moment of Inertia Ip1 | 1.3093e-28 kg·m² |
| Moment of Inertia Ip2 | 1.3093e-28 kg·m² |
| Moment of Inertia Ip3 | 1.303e-28 kg·m² |

| Mesh | |
|---|---|
| Physics Preference | Mechanical |
| Size Function | Adaptive |
| Bounding Box Diagonal | 1.7321e-6 m |
| Average Surface Area | 3.1035e-12 m² |
| Minimum Edge Length | 0. m |
| Element Size | Default |

| Isotropic elasticity | |
|---|---|
| Young's Modulus | 2.7618e+9 Pa |
| Shear Modulus | 3.1197e+10 Pa |

| Order | Harmonic frequency [Hz] |
|---|---|
| 1 | 3.2471e+7 |
| 2 | 5.9663e+7 |
| 3 | 6.3903e+7 |
| 4 | 2.3115e+9 |
| 5 | 2.3119e+9 |
| 6 | 2.312e+9 |
| 7 | 2.7615e+9 |
| 8 | 2.7618e+9 |
| 9 | 2.7619e+9 |
| 10 | 2.7625e+9 |

**Figure S1: Simulation of the harmonic frequency of a magnetic bead with a diameter of 1$\mu$m. The first harmomic frequency is 32.4MHz.**

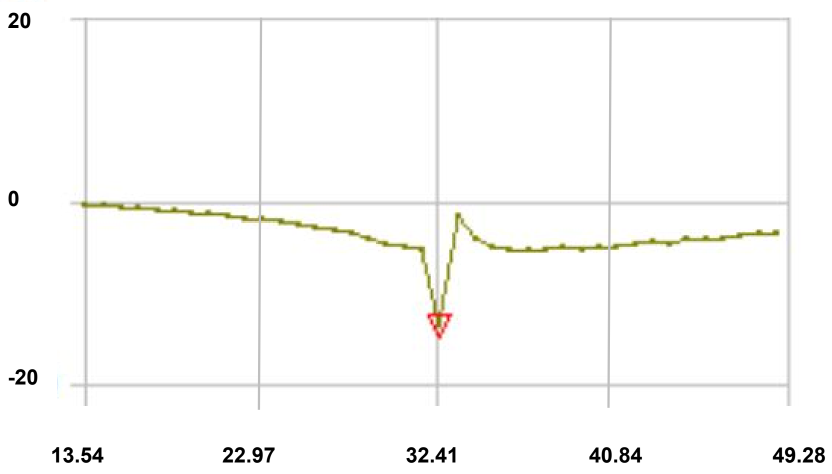


**Figure S2:** The actual central frequency of the IDT that desiged with the harmonic frequency of magnetic beads.

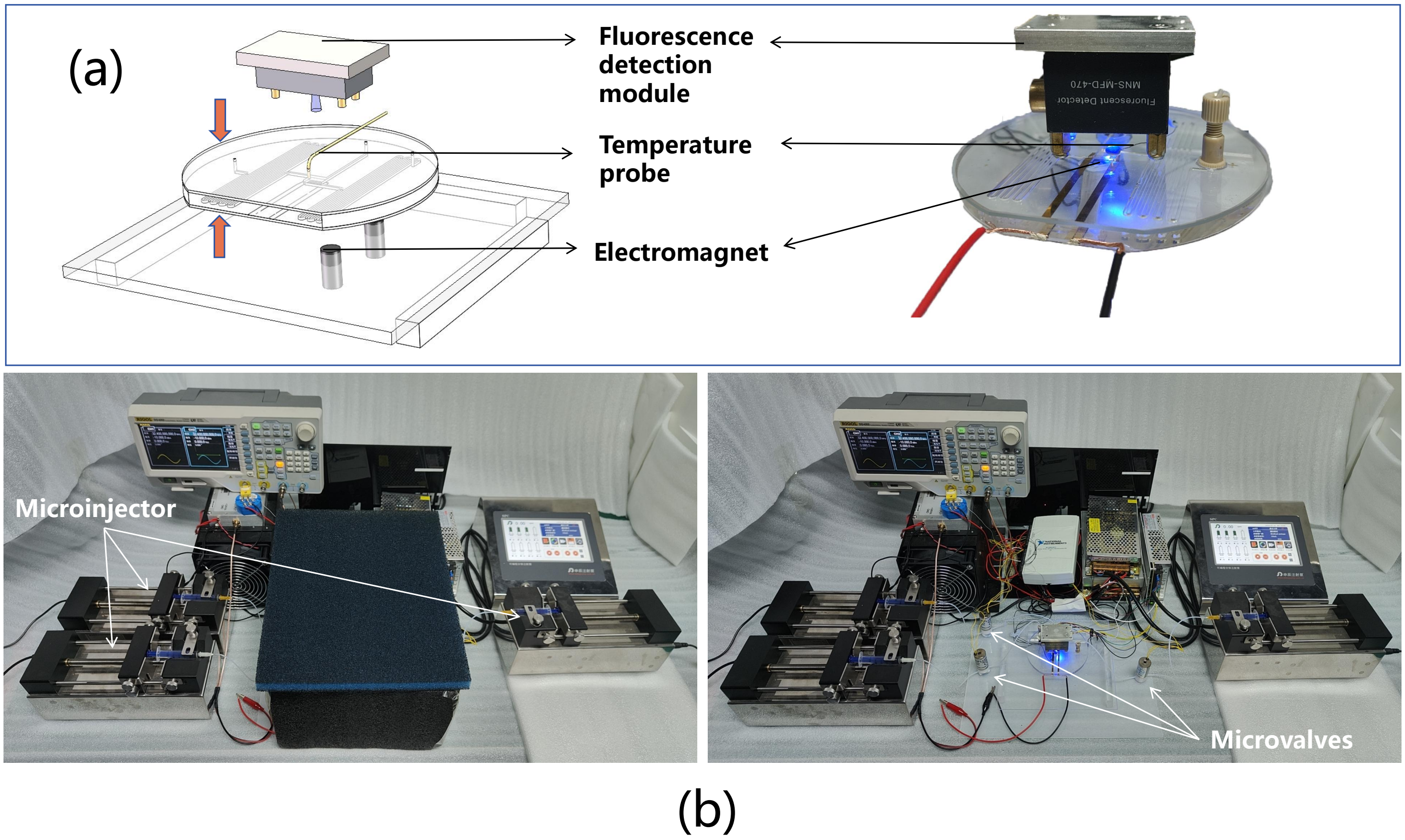


**Figure S3:** (a) RNA detecting device assembly. (b) Experimental set-up of the microfluidic platform.

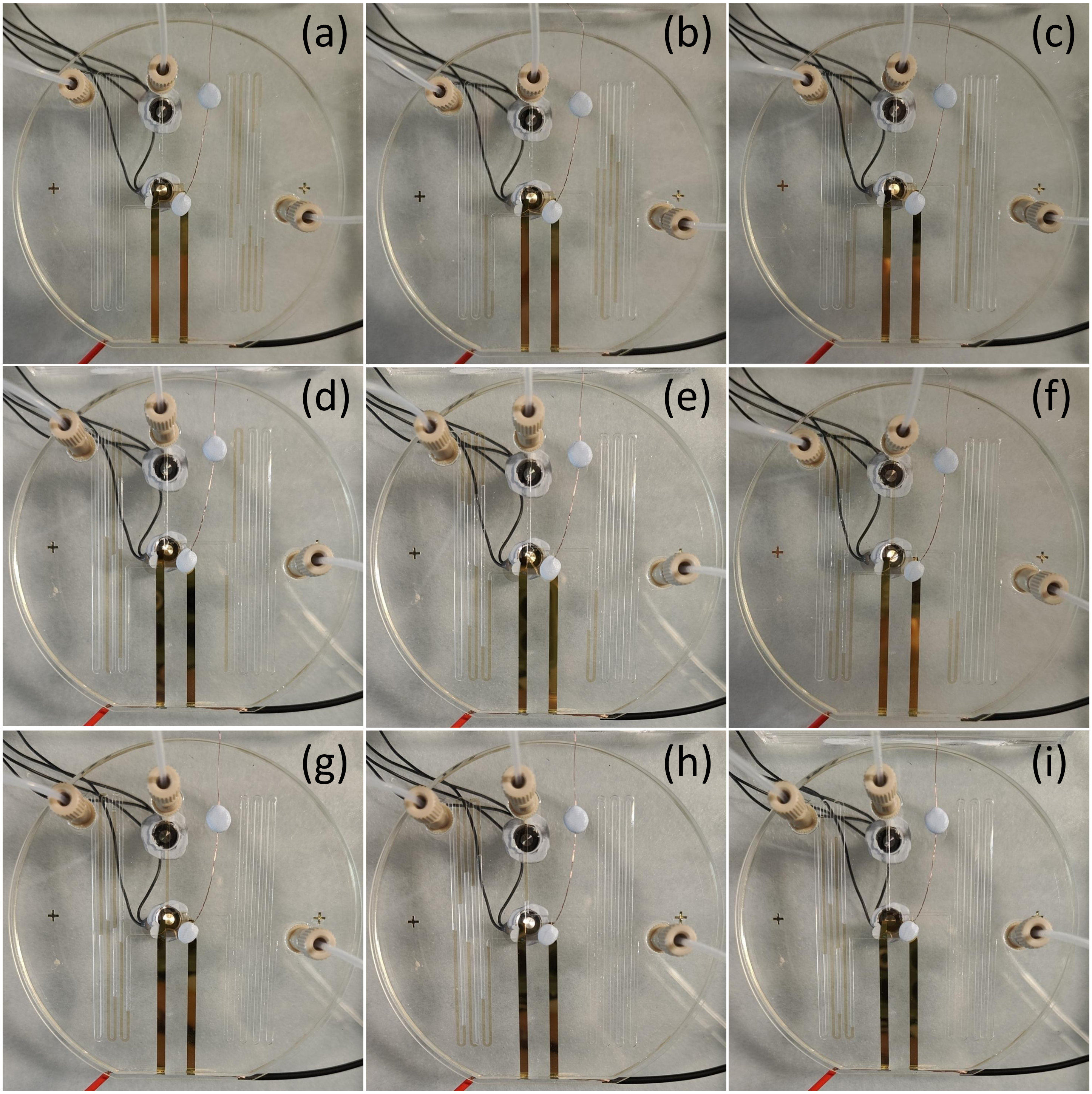


**Figure S4: Workflow of the automatic fluidic control process. (a) Cell lysis by SAW-induced magnetic bead collisions. (b) Washing off the impurities with the washing solution. (c) Washing away DNA and proteins with a purified solution. (d) Repeating the washing process for a second time. (e) Filling eluate into the reaction chamber. (f) Separating magnetic beads with a magnetic field. (g) Mixing RNA with the PCR premix. (h) Blotting off excess RNA samples. (i) SAW-induced PCR thermal cycling.**

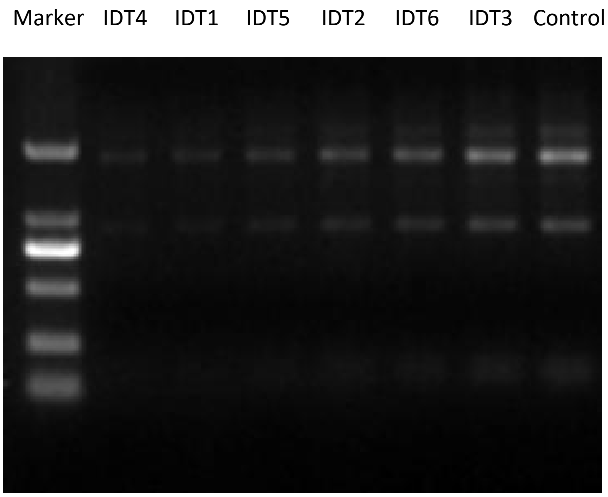


**Figure S5: The gel electrophoresis analysis of six IDTS.**

The frequency of SAW and the ambient pressure were 32.4 MHz and 1.01e-5 Pa, respectively. The simulation results showed that the magnetic beads were suspended in the reaction chamber with the help of SAWs. To observe the suspension of magnetic beads more accurately, we also conducted an experiment and simulation in a water droplet.

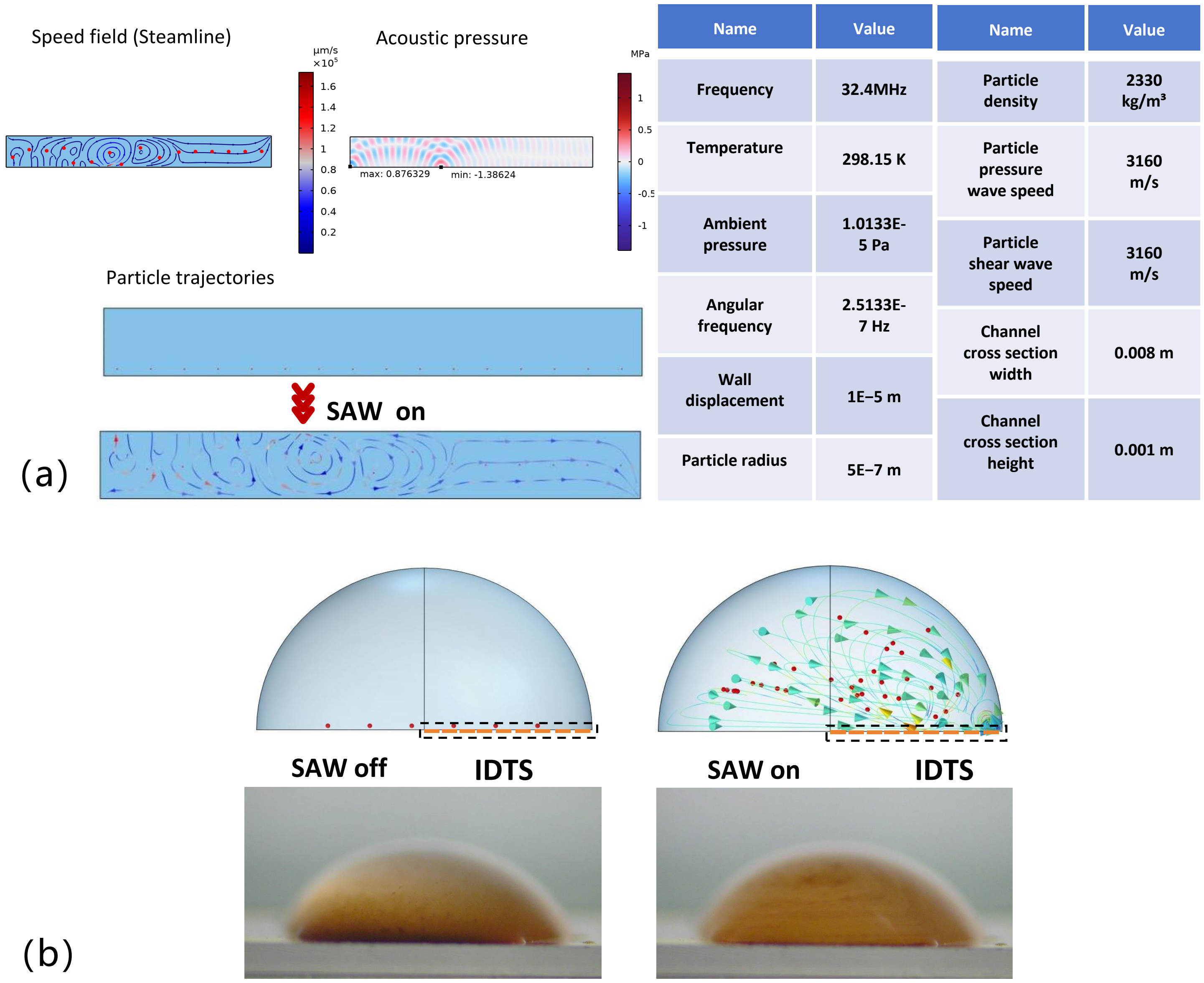


| Name | Value | Name | Value |
|---|---|---|---|
| Frequency | 32.4MHz | Particle density | 2330 kg/m³ |
| Temperature | 298.15 K | Particle pressure wave speed | 3160 m/s |
| Ambient pressure | 1.0133E-5 Pa | Particle shear wave speed | 3160 m/s |
| Angular frequency | 2.5133E-7 Hz | Channel cross section width | 0.008 m |
| Wall displacement | 1E−5 m | Channel cross section height | 0.001 m |
| Particle radius | 5E−7 m | | |

**Figure S6: (a) Motion simulation analysis on magnetic beads in the reaction chamber. (b) Simulation and image of magnetic beads in a water droplet.**

To improve the readability and clarity of the figures, we cropped the original gel electrophoresis graph and rearranged it, allowing readers to easily compare the target result with the control. We also readjusted the size of the image for better layout purposes. To assist with understanding, the positions of the images in Figure 10e are illustrated in Figure S7.

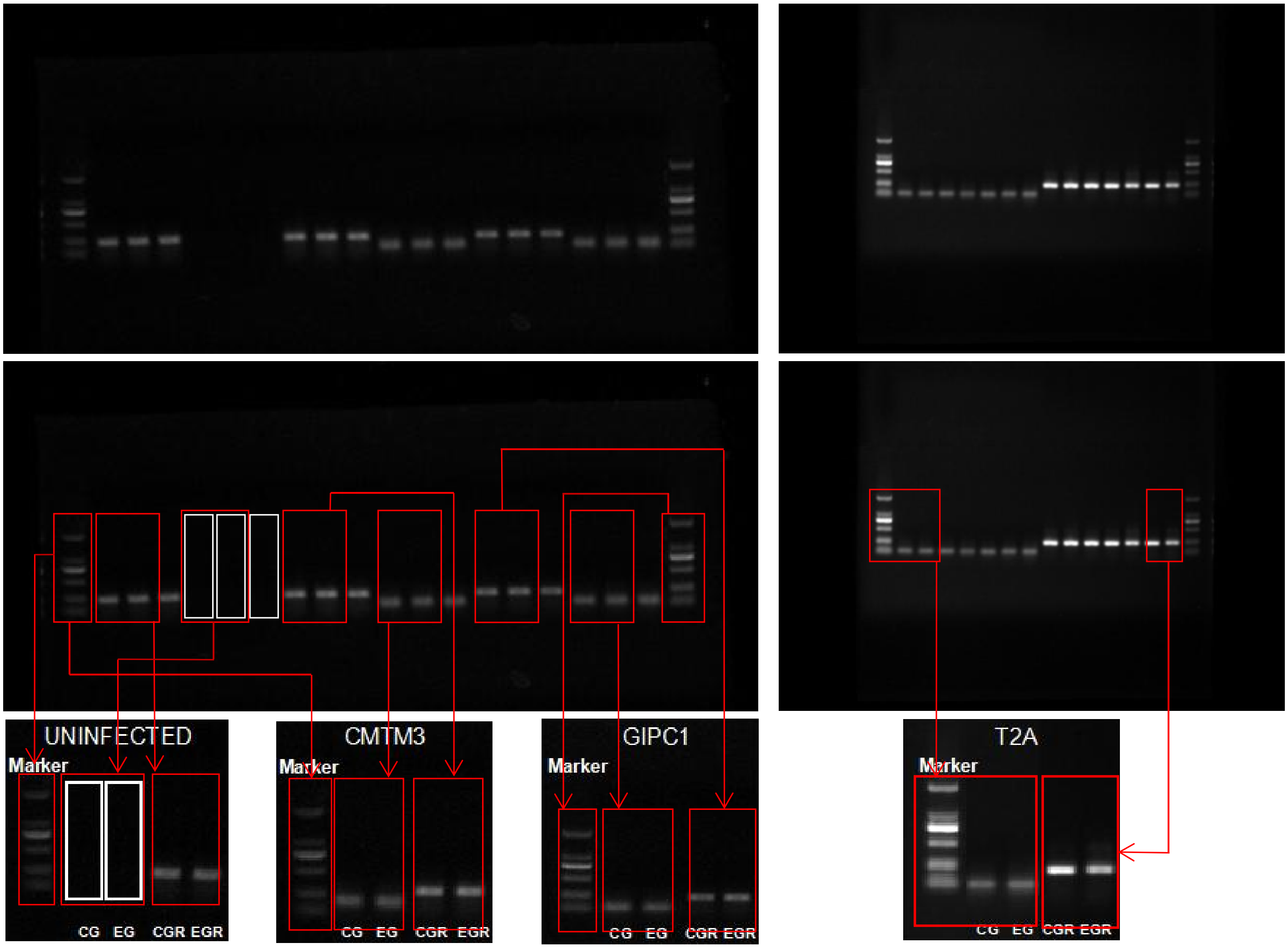


**Figure S7: The positions of the images in Figure 10e.**

# Supplementary Video

Video 1: Video of the workflow of the automatic fluidic control process. As the reagents were transparent, we replaced the reagent with culture medium to improve the video's contrast.
